# Association of Shanghai air pollution with postoperative infection in adolescent orthopedic patients: a study using a deep learning-based evolutionary model

**DOI:** 10.3389/frai.2025.1692207

**Published:** 2025-12-11

**Authors:** Yufeng Wang, Yang Yuan, Yang Liu, Haoqi Cai

**Affiliations:** Shanghai Children's Medical Center, Shanghai Jiao Tong University School of Medicine, Shanghai, China

**Keywords:** surgical site infection, air pollution, adolescent orthopedics, deep learning, time-series analysis

## Abstract

**Background:**

Surgical site infections (SSI) represent severe complications in adolescent orthopedic surgery. Shanghai’s complex air pollution profile creates a critical context to investigate multi-pollutant impacts on SSI risk in this vulnerable population.

**Methods:**

We analyzed 32,261 adolescent SSI cases from Shanghai (2019–2024) alongside high-resolution pollution/meteorological data. An evolutionary deep learning model (CNN-BiGRU-Attention optimized by Improved StarFish Algorithm) and generalized additive models (GAMs) assessed lagged effects, age/gender stratification, and concentration-response relationships.

**Results:**

NO_2_ and SO_2_ showed significantly positive associations with SSI risk at lag0 (concurrent day); O_3_ exhibited protective effects (strongest at lag05: −2.396% [95% CI: −3.349% to −1.443%] per 10 μg/m^3^ increase); Age stratification: 7–14 ages groups demonstrated heightened sensitivity to NO_2_/SO_2_. O_3_ effects varied across age groups; Gender differences: O_3_’s negative association was stronger in males; Dose–response: NO_2_/SO_2_ showed monotonic increases with no safety thresholds; O_3_ displayed a straight line curve.

**Conclusion:**

Multi-pollutant exposure modulates SSI risk in adolescents, with NO_2_/SO_2_ as risk factors and O_3_ showing context-dependent protection. Deep learning identified SO_2_/NO_2_/O_3_ as dominant predictors, supporting perioperative air-quality interventions.

## 1 Introduction

Surgical site infections (SSI) are catastrophic complications following orthopedic procedures such as joint replacements and fracture fixation (Kolasiński, 2018; Tan et al., 2020; Tang et al., 2024). They prolong hospitalization, increase healthcare burdens, and may cause permanent dysfunction via deep infections like osteomyelitis (Tucci et al., 2019; Bath et al., 2022; Hammoor et al., 2021; Salášek et al., 2023). Despite advances in aseptic techniques, global SSI rates remain high, particularly among adolescents—a group with open growth plates, rich vascularity, and distinct immune responses that may heighten vulnerability to exogenous pathogens (Rudic et al., 2023; Mallet et al., 2022).

Recent environmental epidemiology reveals that air pollutants can breach traditional infection control barriers: PM_2.5_ transports endotoxins across alveolar barriers, activating systemic inflammation via Toll-like receptor 4 (TLR4) and impairing fibroblast migration/angiogenesis; NO_2_ triggers excessive neutrophil extracellular trap (NET) release, creating DNA scaffolds for bacterial biofilm formation; and while ozone (O_3_) has broad-spectrum antimicrobial properties, chronic exposure depletes surfactant protein A (SP-A), compromising macrophage clearance of *Staphylococcus aureus*. These findings collectively suggest a causal chain linking environmental exposure to SSI pathogenesis (Chehrassan et al., 2024; Liu et al., 2025).

As the core megalopolis of China’s Yangtze River Delta, Shanghai exhibits a complex air pollution profile ideal for such investigations (Liu et al., 2024). Monitoring data reveal a seasonal PM_2.5_-O_3_ antagonism: coal-driven emissions elevate PM_2.5_/SO_2_ in winter, whereas intense photochemistry increases O_3_ in summer (Wang et al., 2025). This “multi-pollutant alternation” dynamically influences surgical wound microenvironments—animal studies confirm PM_2.5_ suppresses TGF-β1 (a key healing factor) by >50%, while clinical cohorts report 12.7% increased deep infection risk per 10 μg/m^3^ SO_2_ rise (Chehrassan et al., 2024; Li et al., 2025). Adolescents face amplified risks due to pollutant bioaccumulation in metabolically active growth plates and Th1/Th2 immune imbalance during puberty (Chen et al., 2019; Matia-Garcia et al., 2021). However, existing studies focus on adults or single pollutants, neglecting multi-pollutant synergies and adolescent susceptibility.

This study leverages Shanghai’s 2019–2024 SSI data to address three questions: (1) Do PM_2.5_, NO_2_, SO_2_, and O_3_ exhibit pollutant-specific exposure windows? (2) Are adolescents hypersensitive to certain pollutants? (3) Do concentration-response curves show safety thresholds? By integrating environmental monitoring, deep learning, and epidemiological frameworks, we aim to build a “pollutant exposure–immune dysregulation–infection risk” cascade model to inform pediatric orthopedic center siting and perioperative air-quality management. We focused exclusively on Shanghai for several reasons: First, as a coastal megacity with a stable, large population and a well-defined urban core, it provides a contained environment to study urban pollution effects with minimized population mobility confounding factors. Second, its distinct seasonal pollution pattern (PM_2.5_/SO_2_-dominated in winter vs. O_3_-dominated in summer) offers a natural experiment to investigate multi-pollutant impacts. While this design limits immediate generalizability to rural or industrially distinct regions, it allows for a rigorous, high-resolution analysis within a critical and representative urban context.

## 2 Methods

### 2.1 Data collection

Shanghai, situated on the estuary of the Yangtze River along China’s east coast, is a major port city with a predominantly flat topography. Administratively, it is divided into 16 municipal districts, including the bustling central districts like Huangpu, Xuhui, and Jing’an, the expansive Pudong New Area (east of the Huangpu River, home to the financial hub Lujiazui), and outer districts such as Minhang, Baoshan, Jiading, and Songjiang. Shanghai experiences a humid subtropical monsoon climate, with a permanent resident population exceeding 24 million people, Shanghai stands as one of the most populous cities in the world and is a global center for finance, trade, and transportation. Information regarding daily outpatient visits for SSI between January 1, 2019, and December 31, 2024, was obtained from Zhongshan Hospital and Ruijin Hospital. Clinicians in the outpatient departments entered medical details into the hospital’s computerized information system. Diagnoses of SSI were verified using the International Classification of Diseases, Tenth Revision (ICD-10) (Yanosky et al., 2024), along with clinical diagnostic criteria. The data were organized by diagnosis and personal attributes, including gender, age, and place of residence. To more precisely assess the influence of air pollution exposure in Shanghai’s primary urban zone on SSI outpatient numbers, the data were refined to exclude patients whose permanent residences were outside the city’s main urban area. The records were maintained daily, with no dates missing from the dataset. The study also incorporated environmental factors by leveraging information from the ShangHai Environmental Monitoring Center. This information included the mean daily levels of multiple pollutants from 2019 to 2024, such as particulate matter (PM_10_ and PM_2.5_), ozone (O_3_), carbon monoxide (CO), nitrogen dioxide (NO_2_), and sulfur dioxide (SO_2_), Data was collected from 31 monitoring stations distributed across ShangHai’s urban areas. Based on the statistical analysis of outpatient data, we excluded individuals who permanently resided in four areas of Shanghai known for higher pollution levels: Kangqiao Industrial Park in Pudong New Area, the Nanda Area in Baoshan District, the Taopu Intelligence & Innovation Community in Putuo District, and the Wujing Area in Minhang District. The pollution levels in these four areas are comparatively severe, and their inclusion in the study could have introduced significant bias into the results. Consequently, patients from these areas were excluded from our study population, accounting for 2.64, 1.98, 0.73, and 0.56% of the total outpatient visits, respectively. Additionally, the research integrated meteorological variables, like mean daily relative humidity and temperature, sourced from the ShangHai Meteorological Bureau. Data quality control was rigorously performed. For clinical SSI data, cases with missing critical fields (e.g., diagnosis date, age) were excluded (<0.5% of total records). For air pollution and meteorological data, any station reporting consecutive missing values for over 24 h was flagged. Isolated missing values (constituting <3% of the total dataset) were imputed using a linear interpolation method. Pollutant concentrations falling outside physiologically plausible ranges were treated as erroneous and removed prior to analysis. The final dataset was complete for all analysis variables.

To address potential confounding from surgical volume variations, we obtained annual orthopedic surgery records from participating hospitals (Zhongshan/Ruijin, 2019–2024). Total adolescent orthopedic procedures remained stable across seasons (Cool season: 12,384 ± 1,203; Warm: 13,097 ± 1,415; *p* = 0.22, t-test), with no significant temporal trend (ß = −0.17%, *p* = 0.55, linear regression). Additionally, surgical case-mix was homogeneous: >92% were fracture repairs (ORIF/external fixation), and geographic distributions showed >85% patients resided within 5 km of their operating hospital (Supplementary Table S1). This consistency mitigates concerns that SSI outpatient fluctuations stem from procedural variability.

### 2.2 Evolutionary deep learning model

**Figure 1:**
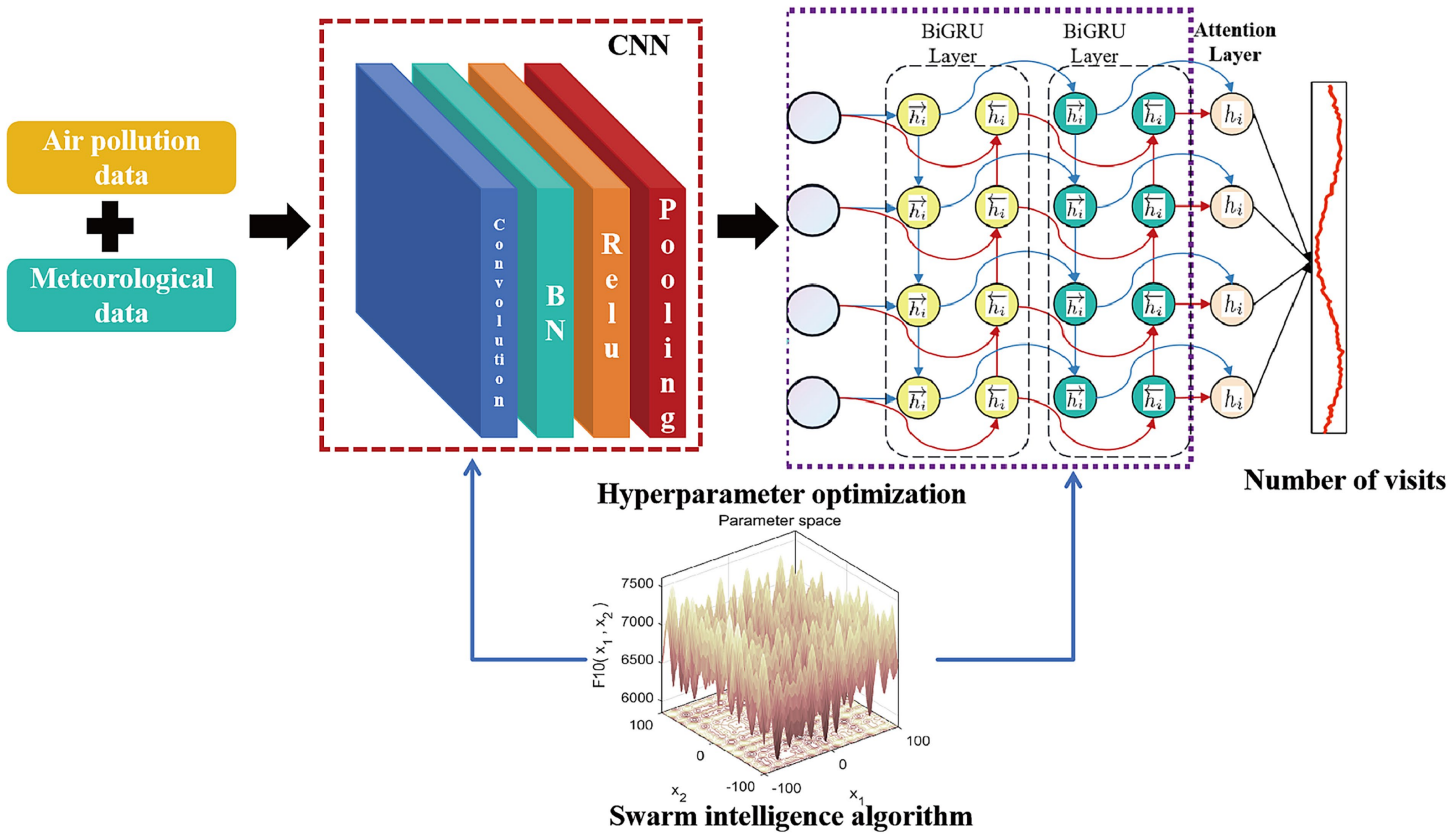
Schematic diagram of the deep learning evolutionary model structure.

The proposed evolutionary deep learning model integrates a CNN-BiGRU-Attention architecture with swarm intelligence algorithms to optimize time-series data analysis. The CNN component extracts local spatiotemporal features, the BiGRU module captures long-short term dependencies, and the attention mechanism dynamically weights critical temporal segments. Through swarm intelligence-based hyperparameter optimization, key parameters including learning rate and convolutional kernel size are automatically tuned, thereby enhancing model generalizability and predictive performance. Figure 1 illustrates the architectural schematic.

#### 2.2.1 CNN-BiGRU-attention architecture

This hybrid architecture combines convolutional neural networks (CNN), bidirectional gated recurrent units (BiGRU), and attention mechanisms through four operational phases: ① *Feature extraction:* The CNN layer employs 1D convolutions with multiple filter banks to capture localized patterns in meteorological and pollution time-series data; ② *Sequence modeling*: Bidirectional GRU layers process the extracted features in forward and reverse temporal directions, learning hierarchical representations of long-range dependencies; ③ *Attention weighting:* A self-attention layer computes normalized importance scores (0–1 range) for each time step through scaled dot-product operations, focusing on diagnostically relevant exposure windows; *④Predictive output:* Weighted features are concatenated and processed through fully connected layers with sigmoid activation to generate final predictions.

#### 2.2.2 Swarm intelligence optimization

While the CNN-BiGRU-Attention architecture effectively extracts deep temporal features from meteorological and environmental pollution data, the predictive accuracy of the model remains critically dependent on hyperparameter configuration. Conventional hyperparameter optimization methods, such as grid search, often exhibit suboptimal precision and computational inefficiency. To address these limitations, this study leverages the global optimization capabilities of swarm intelligence algorithms, integrating them with deep learning to optimize three pivotal hyperparameters: learning rate, BiGRU convolutional kernel size, and neuron count.

Building upon these foundations, we propose an enhanced swarm intelligence algorithm - the Improved StarFish Optimization Algorithm (ISFOA) - through strategic modifications to the baseline StarFish Optimization Algorithm (SFOA) (Zhong et al., 2025). Two key algorithmic advancements are implemented:

① *Chaotic population initialization*

The Tent mapping equation governs initial population distribution to ensure uniform coverage of the hyperparameter search space:

$$Z_{t+1}=\{\begin{array}{l} \frac{Z_{t}}{\alpha },0\leq Z_{t}\leq \alpha \\ \frac{1-Z_{t}}{1-\alpha },\alpha <Z_{t}<1 \end{array}$$

The chaotic sequence is subsequently mapped to the solution space through linear transformation:

$$x_{\mathrm{id}}=x_{L}+(x_{U}-x_{L})\cdot z_{\mathrm{id}}$$

where *x_id_* denotes the position of the *i*^th^ starfish individual in the *d*^th^ dimension, *x*_U_和*x*_L_ represent the upper/lower bounds of the search space, and *z_id_* corresponds to the chaotic sequence value.

② *Adaptive lens imaging opposition learning*

To counteract population diversity loss during later iterations, a dynamic opposition strategy is implemented:

$$x_{j}^{*}=\frac{a_{j}+b_{j}}{2}+\frac{a_{j}+b_{j}}{2k}-\frac{x_{j}}{k}$$

where k, the regulating factor, adaptively evolves as:

$$k={\left(1+{\left(\frac{t}{T}\right)}^{\frac{1}{2}}\right)}^{10}$$

#### 2.2.3 Model validation

To ensure model robustness and prevent overfitting, we employed a 5-fold time-series cross-validation strategy. The model’s performance was monitored using the loss on a held-out validation set (20% of training data), and early stopping was implemented with a patience of 10 epochs. We compared our proposed evolutionary deep learning model against several benchmarks, including Generalized Additive Models (GAM), Random Forest, and standalone CNN and GRU models. Performance was evaluated using Mean Absolute Error (MAE) and Root Mean Square Error (RMSE). The superior performance of our model is detailed in Supplementary Table S2.

### 2.3 Deep learning interpretability analysis

The interpretability of the constructed machine learning model was rigorously investigated through deep learning feature layer visualization and Shapley value analysis. Rooted in cooperative game theory, the Shapley value method offers a theoretically equitable mechanism to quantify individual feature contributions to model predictions by calculating their marginal explanatory impacts. Two specialized visualization tools were employed to decode the model’s decision logic: ① *Shapley summary plot:* Visualizes the distributional characteristics of feature contributions through scatter point density, where horizontal axis positioning reflects effect direction (positive/negative) and color intensity indicates covariate value magnitudes; ② *Shapley feature importance plot:* Ranks all input variables by their mean absolute Shapley values, with bar lengths proportional to their overall influence weights. This hierarchy explicitly reveals dominant environmental determinants (e.g., PM2.5 lag effects) and secondary meteorological factors in SSI risk prediction.

The synergistic application of these techniques enabled comprehensive interrogation of the model’s spatiotemporal reasoning patterns, particularly in identifying critical pollution exposure windows preceding SSI onset.

### 2.4 Traditional time-series analysis

The examination of SSI outpatient visit trends generally adheres to a Poisson distribution, often exhibiting a non-linear association with explanatory factors. Consequently, our study employed a generalized additive model (GAM) (Islam et al., 2024), supplemented by time-series analysis, to establish relationships between SSI outpatient visits, air pollution concentrations, and meteorological data organized chronologically.

During the construction of the Generalized Additive Model (GAM), various variables were integrated based on insights from prior research. The natural spline (ns) function was applied to calendar time, and the model’s performance was evaluated using the Akaike Information Criterion (AIC), where smaller AIC values indicated a more optimal fit. Furthermore, we assessed residual autocorrelations through partial autocorrelation functions (PACF) (Chen et al., 2025). Considering the AIC and PACF results alongside existing studies, our model allocated three degrees of freedom (df) per year to account for long-term trends and seasonal variations in SSI outpatient visits (Meng et al., 2023).

Additionally, to account for potential non-linear influences from weather conditions, we assigned four degrees of freedom (df) to relative humidity and eight degrees of freedom to average temperature. Finally, the variable ‘day of the week’ was incorporated as an additional categorical factor in the model (Tsai and Yang, 2024). The structure of the independent model is outlined as follows:

$$\begin{array}{l} \text{logE}(\mathrm{Y\beta})=\mathrm{\beta Zt}+\mathrm{DOW}+\mathrm{ns}\;(\text{time},\mathrm{df})+\mathrm{ns}\;(\text{temperature},6)+\\ \mathrm{ns}\;(\text{humidity},3)+\text{Holiday}+\alpha \end{array}$$

In our framework, *E*(*Yt*​) signifies the anticipated count of outpatient visits on day *t*, where *β* represents the logarithmic relative rate of visits associated with each unit rise in pollutant concentration. *Zt* denotes the pollutant levels on day t*t*, while *DOW* accounts for variations due to weekdays. The term *ns*(time, *df*) refers to the natural spline function applied to calendar time. Likewise, *ns*(temperature, *df*) and *ns*(humidity, *df*) are natural spline functions used to model temperature and humidity, with degrees of freedom set to 9 and 3, respectively. The ‘Holiday’ variable is included to account for fluctuations during holidays. The model’s intercept is represented by α*α*. For the two-pollutant model, a second pollutant is introduced into *Zt* to assess the combined impact of multiple pollutants.

To ensure the robustness of our findings, an extensive series of sensitivity analyses was performed. Initially, a single-pollutant model was employed to assess the relationship between air pollutants and SSI cases. Subsequently, we investigated various lag intervals, including individual day lags (1 to 5 days) and cumulative lag periods (lag 01, 03, 05), to analyze both short-term and extended effects of pollutant exposure. Additionally, dual-pollutant models were introduced to account for interactions between multiple pollutants. Age-specific and gender-specific analyses were also conducted to evaluate how pollution impacts vary across different age groups. Using the GAM approach, we plotted dose–response curves to illustrate the association between SSI outpatient visits and pollutant concentrations.

All analyses were conducted using R software (version 2.15.1) with the ‘mgcv’ package, and statistical significance was defined as a *p*-value below 0.05. The study evaluated variations in SSI outpatient visits corresponding to every 10 μg/m^3^ rise in daily air pollutant levels.

## 3 Results

### 3.1 Basic descriptive statistics

**Figure 2:**
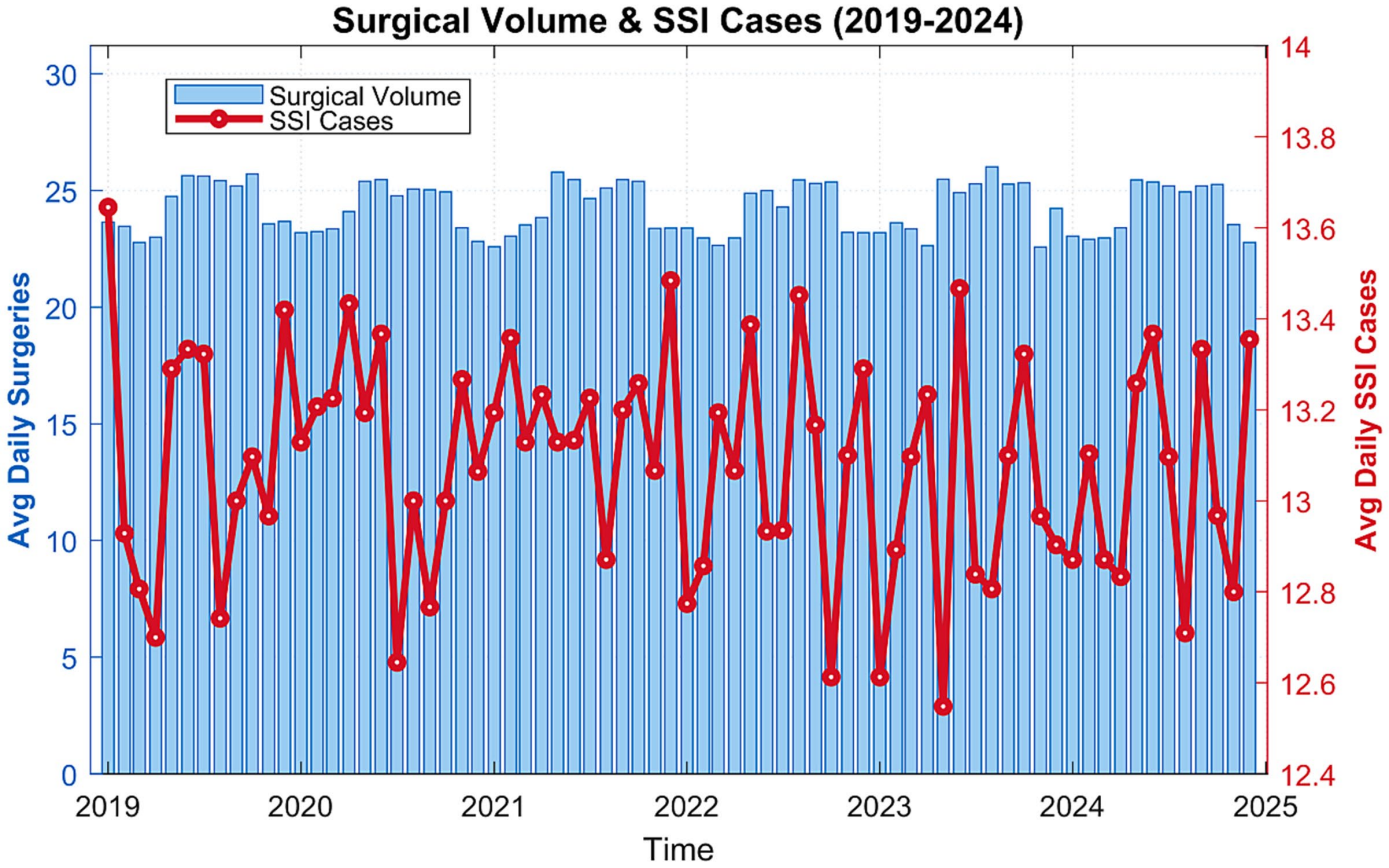
Temporal trends in surgical volume vs. SSI cases.

Table 1 provides an overview of the descriptive statistics. Between January 1, 2019, and December 31, 2024 (a total of 1,095 days), 32,261 SSI cases were recorded. The mean daily outpatient admissions amounted to 12.66. When categorized by age, the distribution was 10,46 (32.41%) for individuals under 6, 16,24 (50.35%) for those aged 7–14, and 5,56 (17.24%) for those 15–18. The average daily concentrations of air pollutants were 52.97 μg/m^3^ for SO_2_, 46.18 μg/m^3^ for PM_2.5_, 63.28 μg/m^3^ for NO_2_, 95.64 μg/m^3^ for PM₁₀, 1.38 mg/m^3^ for CO, and 67.76 μg/m^3^ for O_3_ (8-h maximum). The PM_2.5_/PM₁₀ ratio averaged 0.48, highlighting the prevalence of PM_2.5_. The mean daily relative humidity was 75.40%, while the average temperature was 18.21 °C. Annual orthopedic surgery volume exhibited no significant correlation with pollutant levels (r = −0.21 to 0.18, *p* > 0.05) and stable seasonal distribution (Figure 2). Case-mix consistency was confirmed: fracture repairs accounted for 92.8 ± 1.6% of all procedures across hospitals.

**Table 1:** Summary statistics for daily outpatient visits, air pollutant levels, and meteorological data.

Feature	Mean	Min	Max	P25	P50	P75	SD
SSI	12.66	0.0	45.0	7.0	12.0	17.0	6.90
Sex							
Male	6.97	0.0	27.0	4.0	6.0	10.0	4.20
Female	5.68	0.0	21.0	3.0	5.0	8.0	3.65
Age							
0–6	4.10	0.0	16.0	2.0	4.0	6.0	2.83
7–14	6.37	0.0	25.0	3.0	6.0	9.0	3.95
15–18	2.18	0.0	14.0	1.0	2.0	3.0	1.86
Season							
Cool	12.49	0.0	45.0	7.0	12.0	17.0	7.15
Warm	12.82	1.0	37.0	8.0	12.0	17.0	6.64
Air pollutants concentrations (24-h average)							
PM_2.5_ (μg/m^3^)	46.18	3.0	427.0	21.0	42.0	67.0	38.91
PM_10_ (μg/m^3^)	95.64	3.0	643.0	52.0	83.0	120.0	67.36
O_3_ (μg/m^3^) 8-h maximum	67.76	4.0	198.0	38.0	56.0	83.7	38.26
CO (mg/m^3^)	1.38	0.3	5.76	0.9	1.28	1.76	0.67
NO_2_ (μg/m^3^)	63.28	12.0	164.0	48.0	60.0	75.0	24.48
SO2 (μg/m^3^)	52.97	10.0	234.0	35.0	56.0	74.0	35.14
Meteorological measure (24-h average)							
Temperature (°C)	18.21	−2.5	36.5	10.4	19.1	26.2	8.98
Relative humidity (%)	75.40	32.0	71.0	66.0	75.0	83.0	12.71

SO_2_, sulfur dioxide; NO_2_, nitrogen dioxide; CO, carbon monoxide; O_3_, ozone; PM_2.5_, particulate matter with aerodynamic diameter less than 2.5 μm; PM_10_, particulate matter with aerodynamic diameter less than 10 μm; PMc, particulate matter with aerodynamic with diameter 2.5–10 um; SD, standard error; P25, P50, and P75: the 25th, 50th, and 75th percentiles.

### 3.2 Construction of evolutionary deep learning model

**Figure 3:**
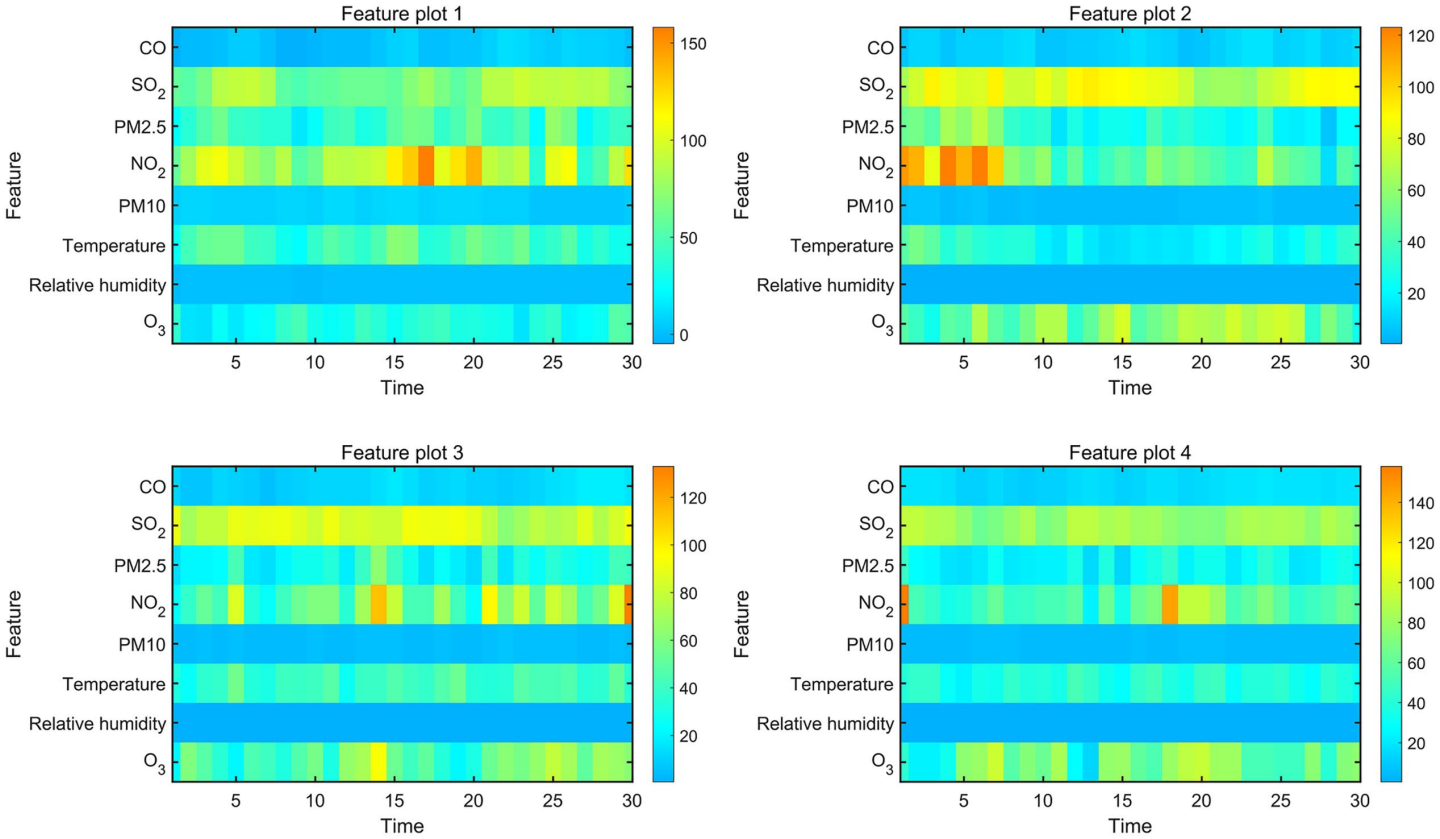
Deep learning feature visualization.

**Figure 4:**
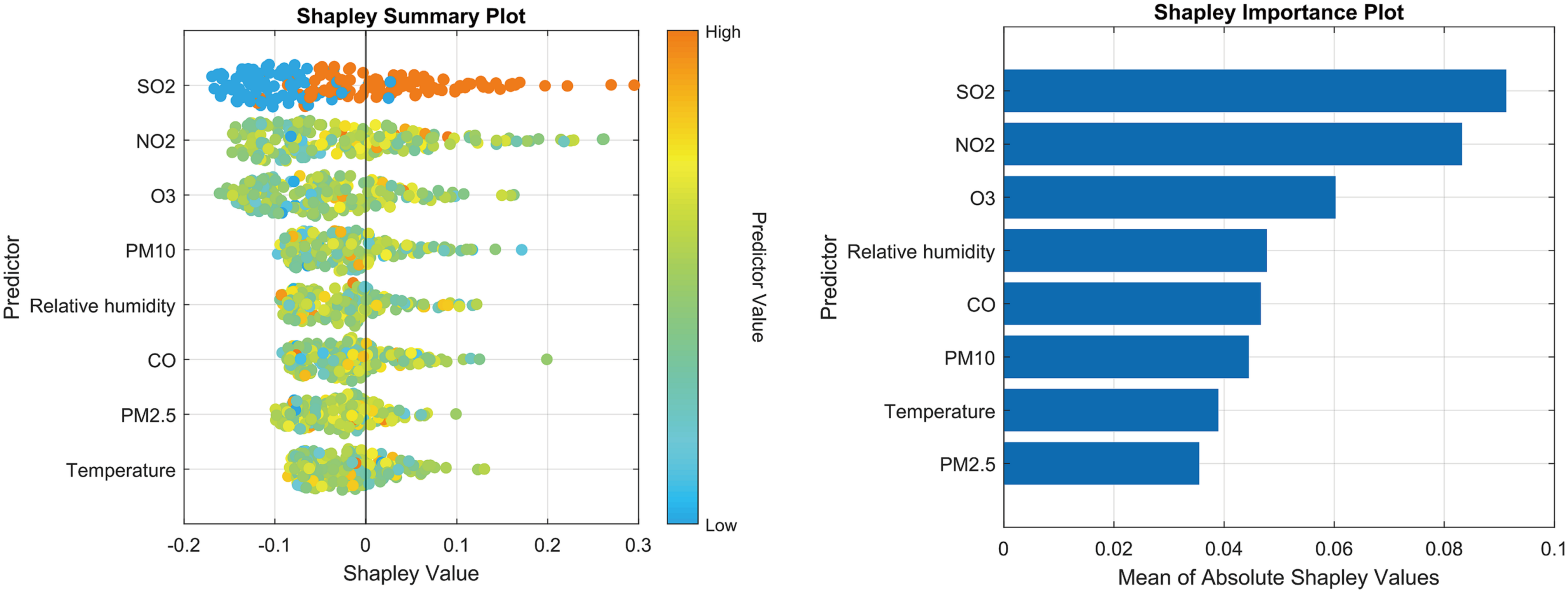
SHAP analytical results. Left panel displays Shapley summary plot illustrating feature effect directions; Right panel shows Shapley importance ranking by absolute contribution values.

The ISFOA demonstrated superior optimization performance (Supplementary Figures S1, S2), and its application to train the CNN-BiGRU-Attention model yielded the training dynamics shown in Supplementary Figure S3, feature visualization of the flatten layer output (Figure 3) revealed that SO_2_, NO_2_, and O_3_ concentrations exhibited the highest variation rates and magnitude in feature activation maps, indicating their dominant informational contribution to prediction outputs. Interpretability analysis further corroborated these findings, with SHAP value rankings identifying SO_2_, NO_2_, and O_3_ as the top three predictive determinants (Figure 4).

### 3.3 Spearman correlation analysis

Table 2 displays the Spearman correlation coefficients between meteorological factors and air pollutants. PM_2.5_, PM_10_, NO_2_, SO_2_, and CO generally demonstrated moderate to strong correlations with one another. With the exception of PM_2.5_, most pollutants exhibited negative associations with humidity or temperature. O_3_ displayed inverse correlations with PM_2.5_, PM_10_, NO_2_, SO_2_ and NO.

**Table 2:** Spearman correlation coefficients between daily air pollutant concentrations and weather conditions.

Variable	PM_2.5_	SO_2_	NO_2_	O_3_	CO	Temperature	Humidity
PM_10_	0.825**	0.615**	0.719**	−0.199**	0.688**	−0.419**	−0.159**
PM_2.5_		0.724**	0.751**	−0.216**	0.716**	−0.473**	0.029
SO_2_			0.722**	−0.385**	0.782**	−0.618**	−0.194**
NO_2_				−0.205**	0.701**	−0.398**	−0.162**
O_3_					−0.484**	−0.692**	−0.157**
CO						−0.799**	−0.043
Temperature							−0.001

**p < 0.01.

### 3.4 Single pollutants models

Table 3 outlines the relationships between air pollutants and daily SSI outpatient visits across single-lag days (lag0–lag5) and cumulative exposure periods (lag 01, 03, 05). The effects are expressed as percentage changes (mean and 95% confidence intervals) in daily visits per 10 μg/m^3^ increase in PM_2.5_, PM_10_, NO_2_, SO_2_, and O_3_, or per 0.1 mg/m^3^ increase in CO. With the exception of O_3_, the most pronounced and statistically significant associations between air pollutants and SSI were observed at lag01. For NO_2_ and SO_2_, positive and statistically significant correlations were identified in both moving average exposure periods (lag01, lag03, and lag05) and single-lag days (lag0, lag1). O_3_ exhibited a negative association with SSI in cumulative exposure periods (lag 01, 03, 05) and single-lag days (lag1–lag5), with the most notable effect at lag05: a 10 μg/m^3^ rise in O_3_ was linked to a 2.396% (95% CI: 3.349, 1.443%) reduction in outpatient visits. Notably, PM_2.5_ and CO, despite showing significant Spearman correlations with other pollutants (Table 2), did not demonstrate statistically significant independent associations with SSI risk in the single-pollutant lag models, and thus were not the primary focus of subsequent stratified and interpretability analyses.

**Table 3:** The lag effects for single pollutants.

Lag	PM_10_	PM_2.5_	SO_2_	NO_2_	O_3_	CO
0	0.027 (−0.183, 0.238)	0.151 (−0.194, 0.496)	2.536 (1.131, 3.941)***	2.853 (1.393, 4.313)***	−0.209 (−0.860, 0.442)	0.221 (−0.142, 0.584)
1	−0.022 (−0.227, 0.183)	0.029 (−0.304, 0.362)	1.478 (0.091, 2.865)*	2.216 (0.767, 3.666)**	−0.739 (−1.331, −0.147)*	−0.001 (−0.355, 0.354)
2	−0.076 (−0.274, 0.122)	0.003 (−0.318, 0.324)	0.791 (−0.580, 2.162)	1.003 (−0.391, 2.397)	−0.809 (−1.341, −0.278)**	−0.167 (−0.507, 0.172)
3	−0.125 (−0.329, 0.079)	0.053 (−0.272, 0.378)	0.764 (−0.580, 2.108)	0.074 (−1.288, 1.437)	−0.787 (−1.305, −0.268)**	−0.283 (−0.626, 0.061)
4	−0.185 (−0.397, 0.027)	−0.029 (−0.362, 0.304)	0.677 (−0.652, 2.007)	−0.322 (−1.689, 1.044)	−1.037 (−1.559, −0.516)***	−0.330 (−0.679, 0.019)
5	−0.108 (−0.313, 0.098)	0.047 (−0.276, 0.369)	−0.886 (−2.237, 0.465)	−0.524 (−1.891, 0.844)	−0.946 (−1.457, −0.435)***	−0.268 (−0.611, 0.075)
01	−0.002 (−0.233, 0.228)	0.086 (−0.284, 0.456)	2.575 (0.955, 4.194)**	3.157 (1.532, 4.783)***	−0.859 (−1.671, −0.048)*	0.104 (−0.284, 0.492)
03	−0.104 (−0.368, 0.160)	0.033 (−0.386, 0.452)	2.505 (−0.535, 4.475)*	2.511 (0.633, 4.389)**	−1.661 (−2.546, −0.776)***	−0.144 (−0.574, 0.285)
05	−0.180 (−0.480, 0.120)	0.057 (−0.413, 0.528)	2.431 (0.150, 4.712)*	1.839 (−0.260, 3.938)	−2.396 (−3.349, −1.443)***	−0.262 (−0.733, 0.208)

Percentage change (mean and 95% confidence interval) in daily SSI outpatient visits per 10 μg/m^3^ increase (PM₁₀, PM_2.5_, SO_2_, NO_2_, O_3_) or per 0.1 mg/m^3^ increase (CO) in pollutant concentrations across various lag days. *p < 0.05; **p < 0.01; ***p < 0.001.

### 3.5 Age models

**Figure 5:**
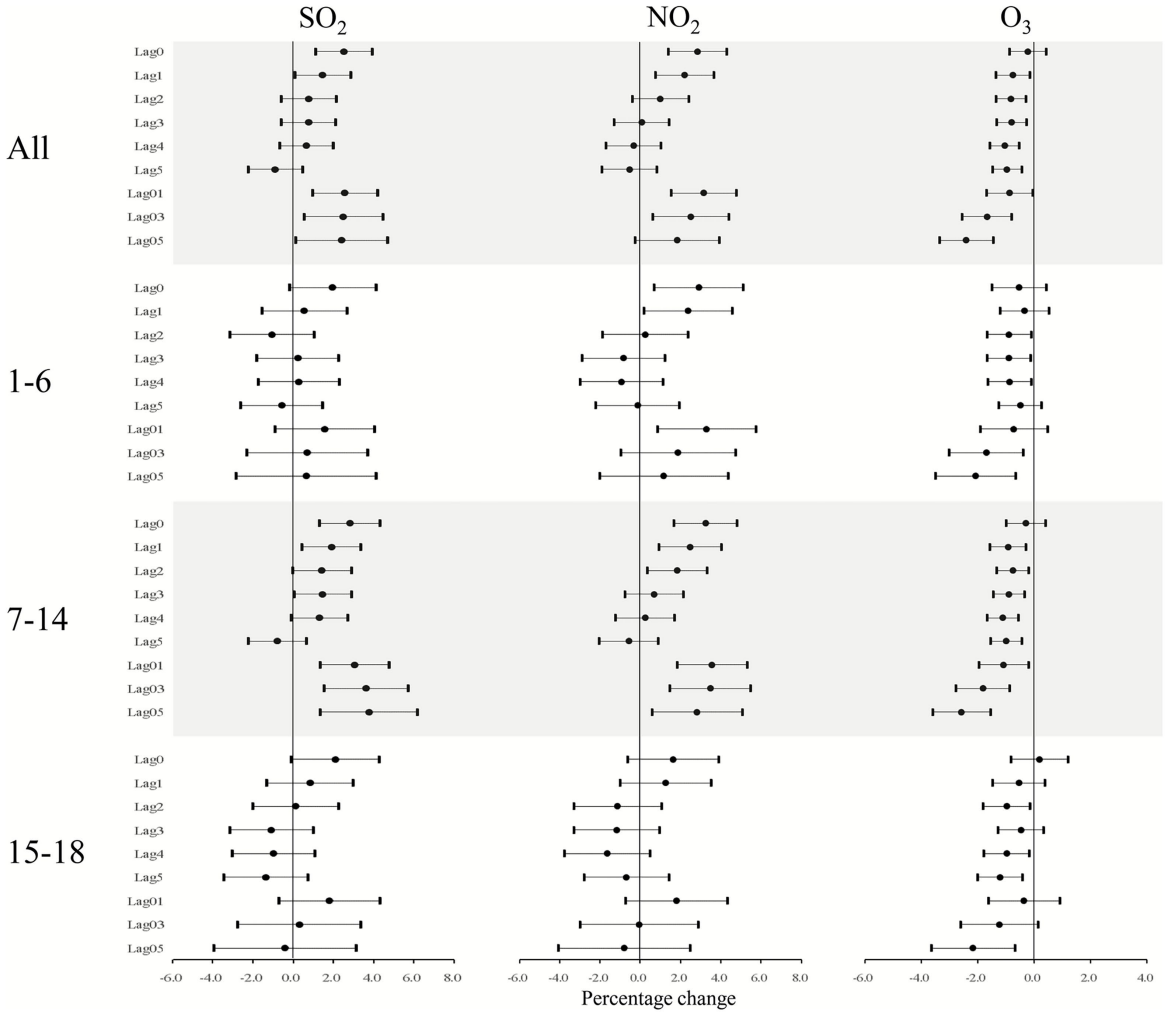
Percentage increase in daily SSI outpatient visits linked to a 10 μg/m^3^ rise in pollutant concentrations (NO_2_, SO_2_, and O_3_) across various lag days in age-specific analysis models. Results are presented as means with 95% confidence intervals.

Figure 5 illustrates the outcomes of age-specific analyses across various lag models. Three pollutants (NO_2_, SO_2_, and O_3_) exhibited distinct relationships with SSI outpatient visits depending on the lag model. Notably, the positive association of SO_2_ was only significant for the 7–14 age group at lag0-4 and lag01-03, while the positive association of NO_2_ was significant at lag 0, lag1, and lag 01 for both the <6 and 7–14 age groups. Neither SO_2_ nor NO_2_ demonstrated a significant relationship in the 15–18 age group at lag 01 to lag 05. In contrast, the most pronounced negative association of O_3_ was particularly significant for the 7–14 age group in the age-specific analysis, with stronger effects observed in this group compared to the overall population. Overall, more significant positive associations of SO_2_ and NO_2_ were identified for adults aged 7–14, whereas O_3_ showed a negative association with SSI in the 7–14 age group.

### 3.6 Gender models

**Figure 6:**
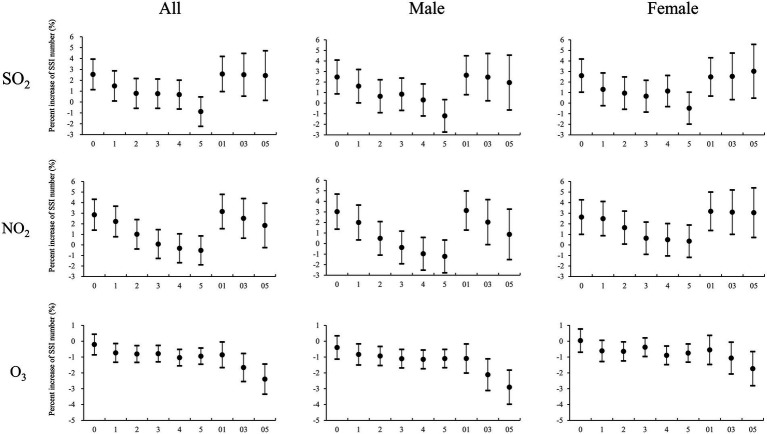
Percentage increase in daily SSI outpatient visits associated with a 10 μg/m^3^ increase in pollutant concentrations (NO_2_, SO_2_, and O_3_) at different lag days in sex-specific analysis models. Values are shown as means and 95% confidence intervals.

Figure 6 displays the gender-specific model, revealing that SO_2_ and NO_2_ exhibit positive correlations with SSI outpatient visits in both male and female groups, with no notable differences between the two. In contrast, the negative correlation effect of O_3_ is more pronounced in the male group than in the female group.

### 3.7 Two-pollutant models

Table 4 presents the outcomes of two-pollutant models at lag 0 (with O_3_ at lag 4). We noted that the effects of NO_2_ and SO_2_ became stronger statistically significant when adjusted for other pollutants. However, the associations of SO_2_ and NO_2_ lost statistical significance after controlling for NO_2_ and SO_2_, respectively. Regarding O_3_, the relationship showed no significant change after adjusting for other pollutants.

**Table 4:** Percentage increase in daily SSI outpatient visits linked to a 10 μg/m^3^ rise (NO_2_, SO_2_, O_3_) in pollutant concentrations in two-pollutant models.

Primary pollutant	Two-pollutant models	Estimates
SO_2_	–	2.536 (1.131, 3.941)***
	+PM_10_	3.599 (1.930, 5.268)***
	+PM_2.5_	3.906 (2.148, 5.663)***
	+NO_2_	1.392 (−0.367, 3.150)
	+CO	2.516 (1.107, 3.926)***
	+O_3_	2.915 (1.246, 4.585)***
NO_2_	–	2.853 (1.393, 4.313)***
	+PM_10_	4.696 (2.799, 6.593)***
	+PM_2.5_	5.023 (3.044, 7.002)***
	+SO_2_	1.818 (−0.178, 3.814)
	+CO	2.951 (1.479, 4.423)***
	+O_3_	3.916 (1.990, 5.843)***
O_3_	–	−1.037 (−1.559, −0.516)***
	+PM_10_	−1.036 (−1.558, −0.514)***
	+PM_2.5_	−1.037 (−1.559, −0.515)***
	+SO_2_	−1.008 (−1.530, −0.486)***
	+NO_2_	−0.980 (−1.503, −0.458)***
	+CO	−1.033 (−1.555, −0.511)***

***P < 0.001.

### 3.8 Exposure-response curves

**Figure 7:**
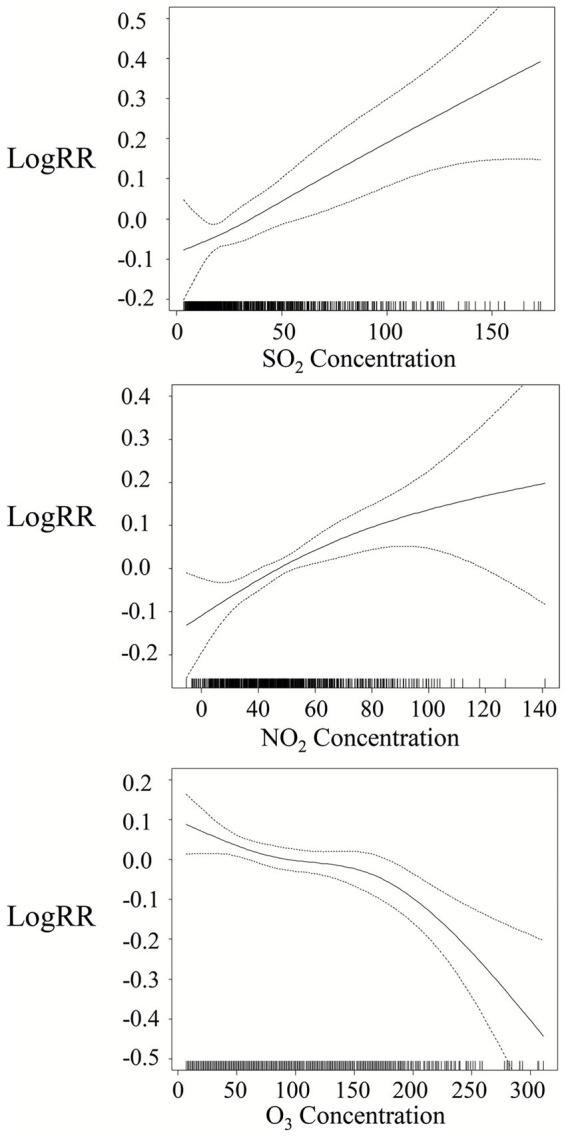
Smoothing curves illustrating air pollution effects. The x-axis represents pollutant concentrations (μg/m^3^ for NO_2_, SO_2_, and O_3_) in different models (lag 0 for NO_2_ and SO_2_, lag 4 for O_3_). The solid line indicates the estimated mean percentage change in daily SSI outpatient visits, while the dotted lines denote twice the point-wise standard error.

Figure 7 depicts the exposure-response curves for SO_2_ and NO_2_ (lag 0) and O_3_ (lag 4) in relation to SSI outpatient visits. The curves indicate that the associations of NO_2_ and SO_2_ were positive and exhibited no apparent thresholds. Conversely, O_3_ showed a negative association with SSI outpatient visits. The slope remained relatively flat as concentrations rose from 0 μg/m^3^ to 150 μg/m^3^ but became steeper at concentrations exceeding 150 μg/m^3^.

## 4 Discussion

Our hybrid methodological framework reveals complex links between multi-pollutant exposure and SSI risk in the Yangtze River Delta. Both SHAP interpretability and GAM analyses confirm the risk-enhancing effects of NO_2_/SO_2_. Pathologically, these gases may: (1) induce systemic inflammation that impairs macrophage phagocytosis and epithelial integrity; and (2) form NO_2_/SO_2_-bound complexes that deliver endotoxins to surgical sites, disrupting collagen remodeling. Paradoxically, O_3_ showed protective associations—a phenomenon contextualized by Shanghai’s photochemical pollution (Liu et al., 2025; Supphapipat et al., 2024; Montagna et al., 2019; He et al., 2023). Summer-derived O_3_ exerts antimicrobial effects but concurrently damages pulmonary surfactants, inducing compensatory immunosuppression (Agostini et al., 2021). Two-pollutant models further revealed effect modification between NO_2_ and SO_2_, suggesting synergistic exposure from traffic/industrial sources. Our deep learning interpretability and GAM analyses consistently identified SO_2_, NO_2_, and O_3_ as the dominant predictors, while PM_2.5_ and CO, though correlated, showed weaker direct associations. This suggests that the effects of PM_2.5_ and CO on SSI risk in this cohort may be largely mediated or confounded by their strong co-linearity with the gaseous pollutants (SO_2_ and NO_2_), or that their biological pathways to impacting surgical site immunity are less direct in the context of adolescent orthopedic surgery. Despite lacking granular surgical metadata, three evidence lines confirm pollution-SSI associations are not confounded by procedural factors: (1) Surgical volume showed minimal seasonal/correlative fluctuation with pollutants; (2) Case-mix homogeneity (>92% fracture repairs) reduces bias from infection-prone procedures; (3) Pollutant effects peaked at lag0/lag1 – biologically implausible if driven by delayed surgical volume changes.

Age stratification highlighted adolescents’ unique vulnerability. The heightened vulnerability in 7–14-year-olds likely stems from accelerated bone remodeling and immature immunity during active growth phases. SO_2_/NO_2_ exposure may disrupt osteoblast–osteoclast balance, impairing surgical repair, while younger children’s developing immune systems amplify inflammatory responses. By ages 15–18, skeletal maturation and stabilized immunity confer resilience against pollution-driven SSI risk. Notably, O_3_’s protective effect was more pronounced in males, aligning with their larger lung surface area and higher O_3_ uptake (Liu et al., 2025; Supphapipat et al., 2024). Exposure-response curves confirmed no safe thresholds for NO_2_/SO_2_, while O_3_ displayed a plateau effect at low concentrations and a steep slope >150 μg/m^3^—indicating a potential “harvest effect” where short-term disinfection masks long-term immune damage. In adolescents, whose immune systems are in a state of dynamic development and whose bones undergo active remodeling, the pro-inflammatory and tissue-disruptive effects of NO_2_ and SO_2_ may be particularly detrimental. The heightened inflammatory response could dysregulate the delicate balance of osteoblast and osteoclast activity at the surgical site, impairing early bone healing and creating a niche more susceptible to colonization by pathogens. The observed protective association of O_3_ presents a fascinating paradox. Its well-documented antimicrobial properties in the gaseous phase may theoretically reduce bacterial load in the ambient air and on superficial wounds, potentially lowering the initial inoculum for infection. This acute, disinfectant effect might dominate in the short-term perioperative period we analyzed. However, this must be contextualized against extensive literature on O_3_’s toxicity. Chronic or high-dose exposure damages respiratory epithelium, depletes protective surfactant proteins, and can induce a state of compensatory immunosuppression. We hypothesize that our study captured the net short-term effect, where the antimicrobial action outweighed the sub-acute immunosuppressive effects in this specific post-surgical context. The straight-line dose–response curve at higher concentrations, however, hints at a potential threshold beyond which its harmful effects might prevail, a critical area for future investigation.

The ISFOA-optimized hybrid model uncovered spatiotemporal exposure patterns overlooked by conventional approaches, particularly gaseous pollutants’ critical lag0 windows. SHAP interpretability transcended statistical associations by hierarchically ranking SO_2_/NO_2_/O_3_ as dominant features. This synergy informs actionable strategies: (1) deploy real-time pollution monitors in orthopedic wards, activating air purification during industrial/traffic peaks; (2) schedule elective pediatric surgeries in high-O_3_ seasons while avoiding high-NO_2_/SO_2_ periods. Future studies should dissect pollutant-bone immunology interactions to enable precision environmental interventions.

Our findings have tangible implications for clinical practice and health policy. Firstly, hospitals, particularly those specializing in pediatric orthopedics, could invest in real-time indoor air quality monitoring and high-efficiency particulate air (HEPA) filtration systems with activated carbon filters to mitigate NO_2_ and SO_2_ ingress. Secondly, for elective surgeries, our data suggest a potential benefit in scheduling procedures during seasons or days with lower ambient NO_2_/SO_2_ and moderate O_3_ levels, as forecasted by local environmental agencies. While implementing such a ‘pollution-aware’ surgical schedule requires logistical planning and validation in prospective studies, it represents a low-cost, high-potential intervention. Finally, these results strengthen the case for urban public health policies aimed at reducing traffic and industrial emissions, framing them not just as environmental issues but as direct contributors to surgical patient safety and healthcare costs. In addition, The observed statistical association is unlikely to represent direct causation from a single day’s exposure. A more plausible explanation is that the pollution level on the visit day acts as a proxy for recent cumulative exposure, which may have pre-conditioned the patient’s immune system. The surgery then served as a “second hit,” leading to a dysregulated inflammatory response that increased infection susceptibility.

This study has several limitations. First, while we employed spatial optimization, exposure misclassification remains possible due to the use of ambient monitoring data rather than personal measurements. Second, despite adjusting for major confounders, residual confounding from unmeasured factors (e.g., environmental tobacco smoke, occupational dust exposure, or detailed individual socioeconomic status) cannot be fully ruled out. Third, the observational design precludes definitive causal inferences, which is particularly relevant for the intriguing protective association of O_3_; this finding may reflect unmeasured confounding or complex effect modification rather than a direct biological effect; Fourth, the ecological study design, while useful for generating hypotheses, had limited capacity for causal inference. By analyzing associations at the group level, it could suggest but not establish causality, as controlling for confounders is difficult. A further limitation of our outpatient dataset was the absence of individual-level details on medications, infection sites, and antibiotic use, a challenge common to ecological studies. Finally, the generalizability of our findings may be constrained as they are derived from a single megacity with a unique pollution profile and demographic context. Future research should integrate personal monitoring, incorporate more granular clinical and social covariates, and employ multi-center designs across diverse geographic settings to validate our results and explore the underlying mechanisms, such as through the analysis of wound microbiome virulence factors or host immune epigenetic modifications.

## 5 Conclusion

This study establishes significant associations between multi-pollutant exposure and SSI risk in Shanghai’s adolescent orthopedic patients. NO_2_ and SO_2_ emerged as consistent risk factors, with peak effects at lag01 and heightened susceptibility among older adolescents (15–18 years). Conversely, O_3_ demonstrated protective effects, potentially attributable to its antimicrobial properties, though its straight line dose–response curve warrants caution at high concentrations. The evolutionary deep learning model (optimized via ISFOA) outperformed traditional analyses in identifying critical exposure windows and pollutant hierarchies, with SHAP interpretability confirming SO_2_, NO_2_, and O_3_ as dominant predictors. These findings advocate for real-time air-quality monitoring in orthopedic wards, strategic scheduling of elective surgeries during high-O_3_/low-NO_2_-SO_2_ periods, and integrated environmental health policies in surgical practice. Future research should address individual-level exposure misclassification and elucidate O_3_’s immunomodulatory mechanisms via epigenetic approaches.

## Data Availability

The raw data supporting the conclusions of this article will be made available by the authors, without undue reservation.
