# Supplementary material for: Association of Shanghai air pollution with postoperative infection in adolescent orthopedic patients: a study using a deep learning-based evolutionary model
Source: Front Artif Intell. 2025 Dec 11;8:1692207. doi: 10.3389/frai.2025.1692207 (PMC12738831; doi:10.3389/frai.2025.1692207)
Supplement: Supplementary file 1 [file Supplementary_file_1.docx]

**Supplementary Material**

**1 Procedural Homogeneity and Localized Patient Base**

surgical case-mix was homogeneous: >92% were fracture repairs (ORIF/external fixation), and geographic distributions showed >85% patients resided within 5 km of their operating hospital (Suppl. Table S1). This consistency mitigates concerns that SSI outpatient fluctuations stem from procedural variability.

Table S1. Surgical Volume Stability

| Year | Annual Surgeries | Cool Season Surgeries | Warm Season Surgeries | Fracture Repair Percent |
| --- | --- | --- | --- | --- |
| 2019 | 8632 | 4094 | 4538 | 93.5779 |
| 2020 | 8715 | 4532 | 4183 | 94.9341 |
| 2021 | 8920 | 3925 | 4995 | 94.6896 |
| 2022 | 9140 | 4601 | 4539 | 94.8795 |
| 2023 | 9321 | 4834 | 4487 | 92.6808 |
| 2024 | 9417 | 4897 | 4520 | 92.3714 |

**2 Performance of model**

Table S2. Performance Comparison of Different Predictive Models

| Model Type | Model Name | MAE  (Mean ± SD) | RMSE  (Mean ± SD) | R²  (Mean ± SD) |
| --- | --- | --- | --- | --- |
| Traditional Model | GAM (Generalized Additive Model) | 2.15 ± 0.23 | 2.89 ± 0.31 | 0.621 ± 0.045 |
| Machine Learning | Random Forest | 1.98 ± 0.19 | 2.71 ± 0.28 | 0.665 ± 0.038 |
|  | XGBoost | 1.87 ± 0.18 | 2.64 ± 0.26 | 0.683 ± 0.036 |
| Deep Learning | CNN | 1.76 ± 0.21 | 2.48 ± 0.29 | 0.714 ± 0.041 |
|  | BiGRU | 1.72 ± 0.17 | 2.42 ± 0.25 | 0.728 ± 0.033 |
|  | CNN-BiGRU (w/o Attention & ISFOA) | 1.65 ± 0.15 | 2.35 ± 0.24 | 0.745 ± 0.030 |
| Proposed Model | CNN-BiGRU-Attention (ISFOA-Optimized) | 1.48 ± 0.12 | 2.14 ± 0.20 | 0.792 ± 0.025 |

**3 Improved Swarm Intelligence Algorithm**

To verify the optimization capability of the improved ISFOA algorithm, this study conducted comparative tests with the original SFOA, WOA, and PSO algorithms. The test functions selected the latest CEC2022 test set, comprising 12 functions. The variable dimension was set to 10, population size to 30, maximum iterations to 500, with 30 independent runs. By comparing the results of 30 runs, box plots were drawn to compare the optimization performance of each algorithm. Results show that ISFOA exhibited superior performance in test functions, demonstrating higher stability compared to the original SFOA and the other two novel algorithms (Fig. S1). Furthermore, convergence curves indicate that ISFOA achieves faster convergence speed with the lowest risk of falling into local optima (Fig. S2).


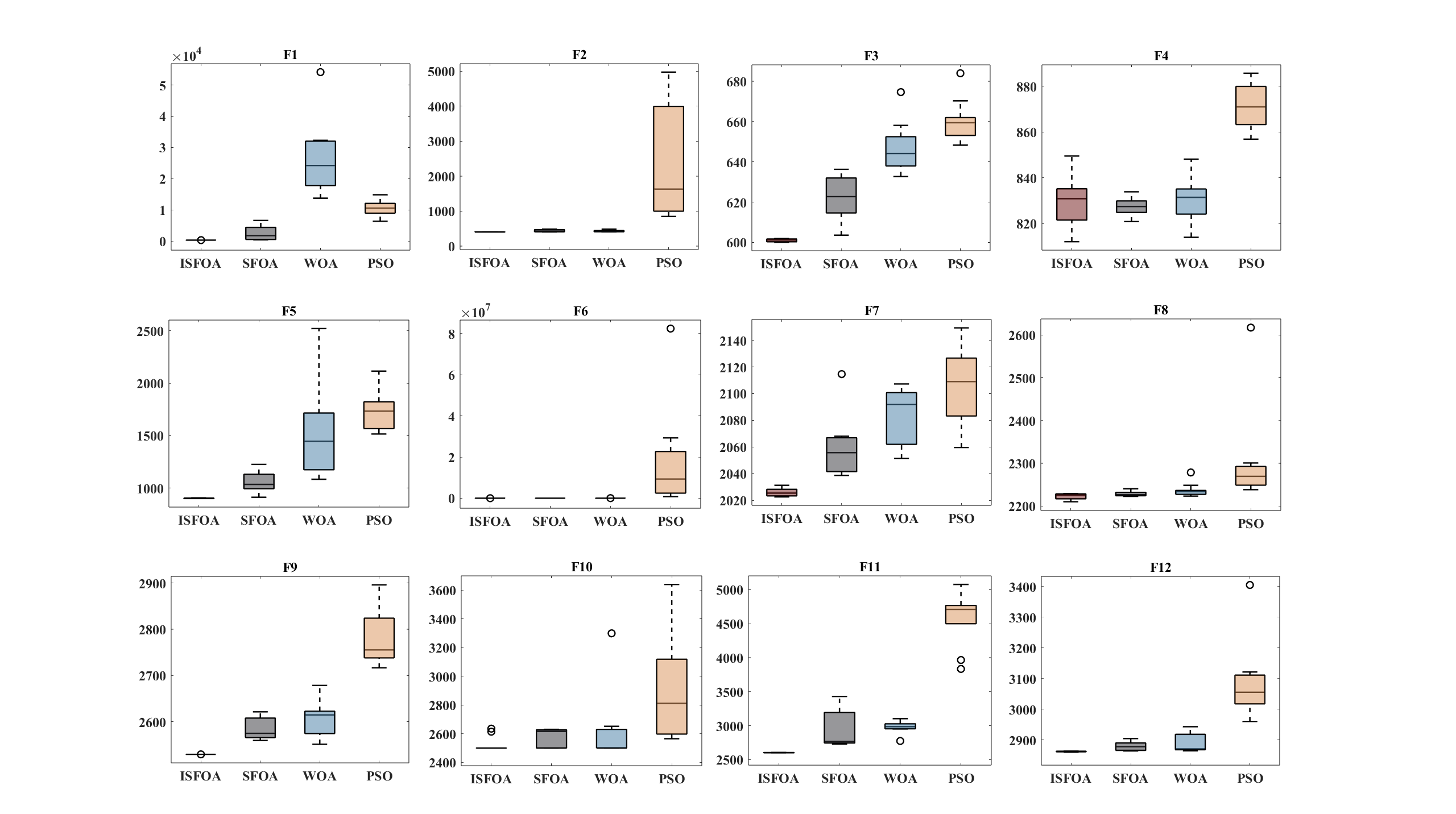


Figure S1 Box plot comparison of optimization results from 30 runs of each swarm intelligence algorithm


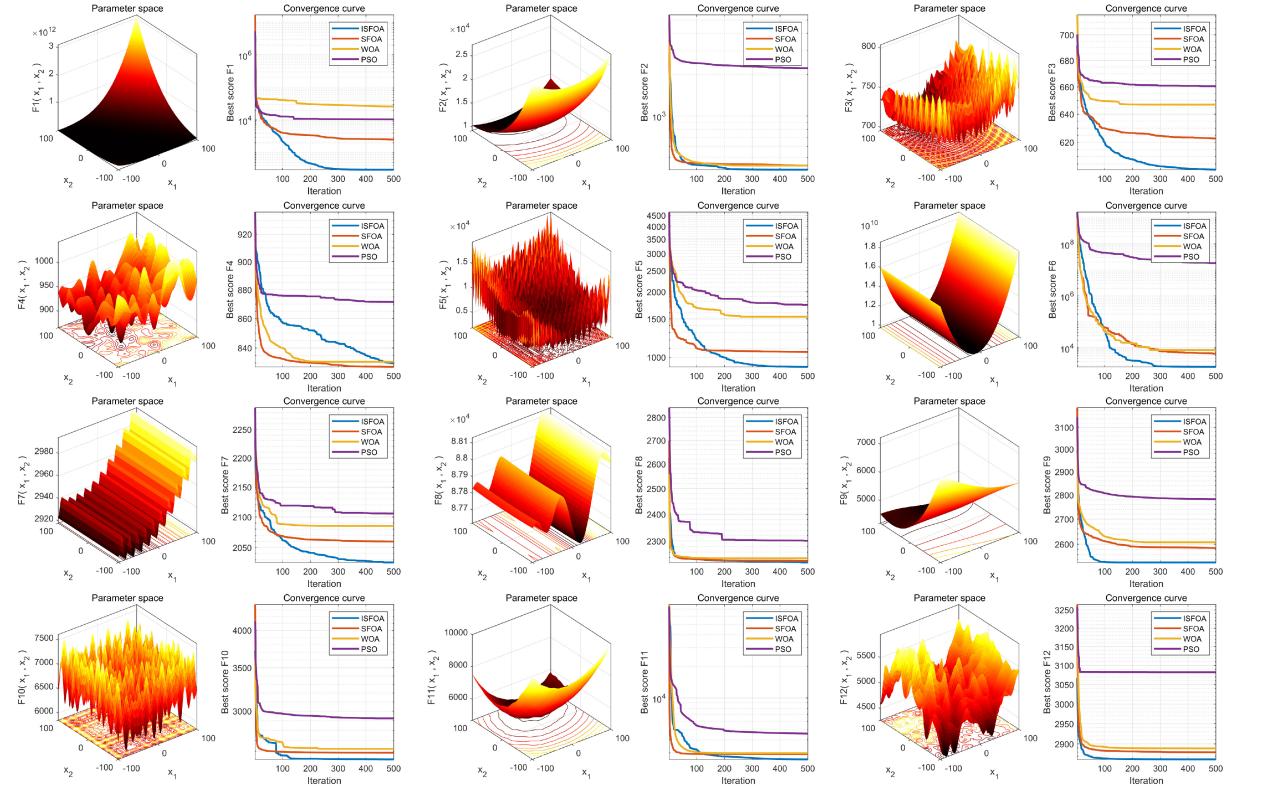


Figure S2 Convergence curves of optimization process for each swarm intelligence algorithm over 30 runs

**4 Model Training Process**

The improved SFOA was utilized to optimize the CNN-BiGRU-Attention model. The objective function was set to RMSE, with iterations set to 10, population size to 4. The optimization range for learning rate was [0.001, 0.01], for convolutional kernel size [2, 5], and for number of neurons [100, 120]. The final model successfully converged (Fig. S3). Optimized parameters were: learning rate: 0.006674201, convolutional kernel size: 5, number of neurons: 101.


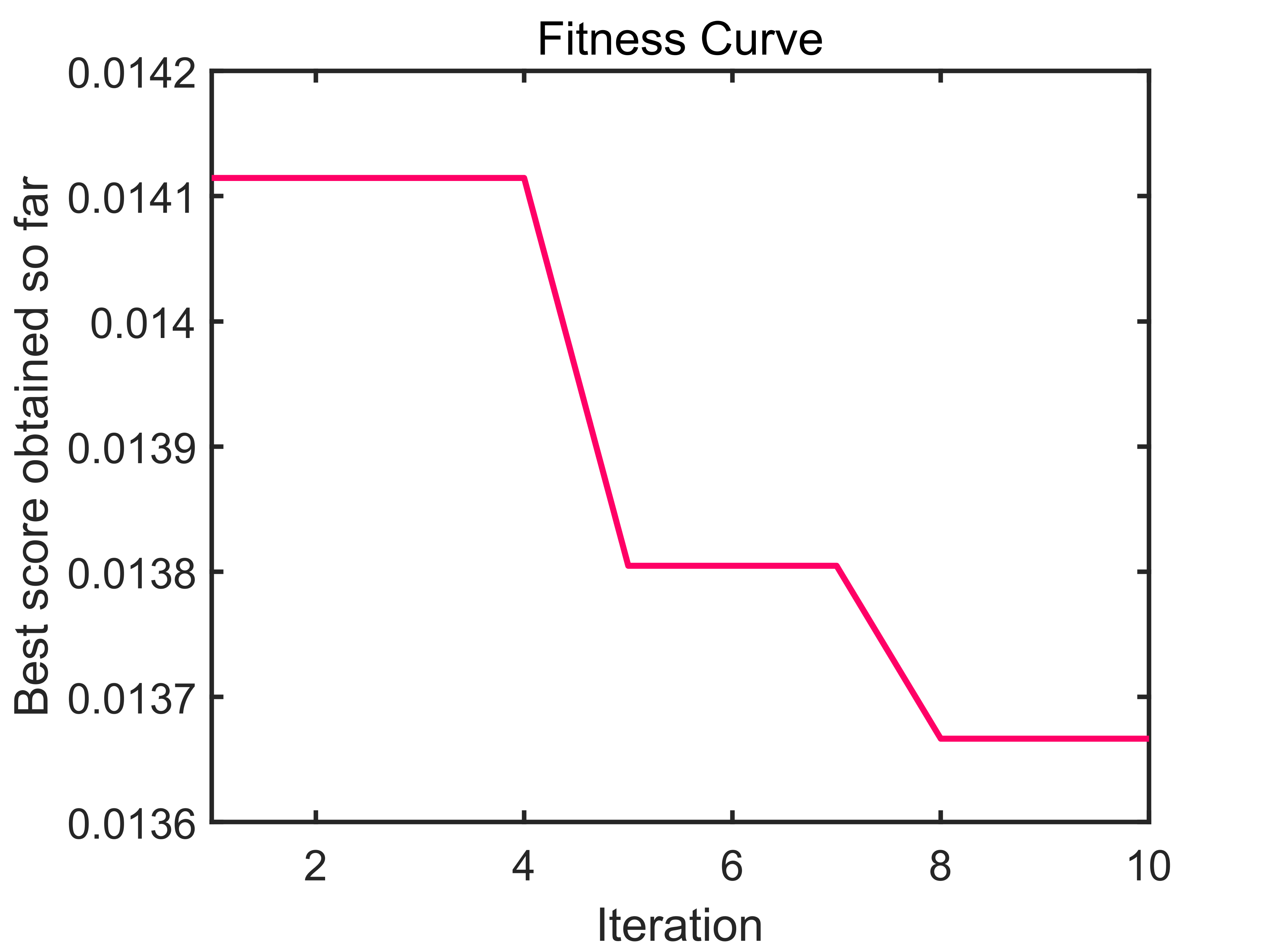

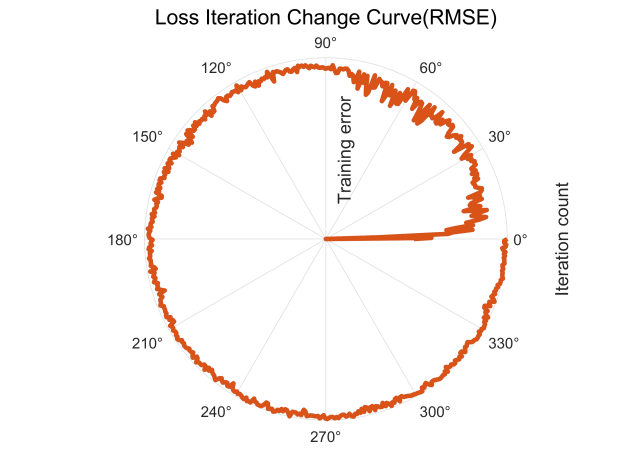


A B

Figure S3 Model training convergence curves

Note：A：Fitness loss convergence curve; B：Polar coordinate loss iteration curve.
